# C-854-01. A Full-coverage Approach to Infection Prevention in Burn Patients: A Pilot Study

**DOI:** 10.1093/jbcr/irag033.077

**Published:** 2026-04-06

**Authors:** Tracy Larson, Pamela Michelli, Howard G Smith, Susan L Smith, Laura E Carpenter, Lauren A Caballero, Brooke Bozoian, Matthew Amacher, Sherrina Richards

**Affiliations:** Orlando Health, Orlando, FL; Advent Health, Orlando, FL; Warden Burn Center at Orlando Health Orlando Regional Medical Center, Orlando, FL; Warden Burn Center at Orlando Health Orlando Regional Medical Center, Orlando, FL; Warden Burn Center at Orlando Health Orlando Regional Medical Center, Orlando, FL; Warden Burn Center at Orlando Health Orlando Regional Medical Center, Orlando, FL; Orlando Regional Medical Center, Orlando, FL; Orlando Health, Orlando, FL; Warden Burn Center at Orlando Health Orlando Regional Medical Center, Orlando, FL

## Abstract

**Introduction:**

Burn wounds are a significant source of infection for patients admitted to an inpatient unit for burn injury. The Centers for Disease Control and Prevention (CDC) [1] reports approximately 3% of all hospital inpatients develop a healthcare-associated infection (HAI), whereas a study of 165 burn patients demonstrated a 27.9% HAI incidence [2]. Decolonization is a proven method to reduce infection caused by organisms including multidrug-resistant organisms (MDROs) [3]. Many patients are decolonized using chlorhexidine gluconate-based (CHG) products. However, even brief exposure of CHG to open burn wounds can result in significant, progressive, and persistent cytotoxicity that results in prolonged wound healing [4]. PH balanced cleanser (PHBC) is a proven alternative that has been shown to be safe over open wounds [5]. The goal of this pilot study is to evaluate the effectiveness of PHBC on the occurrence of infections in adult burn patients admitted to a single verified burn center.

**Methods:**

Institutional review board (IRB) approval was sought before implementation. The pilot study was determined to not meet criteria for human subject research. A protocol was developed for application of PHBC to burns, grafts, intact skin, and skin substitutes in patients admitted to a burn trauma stepdown unit. Electronic medical records (EMRs) before and after were compared for occurrence of infections in samples taken from tissue, respiratory secretions, urine, or blood that were collected for diagnostic or treatment purposes.

**Results:**

In a ten-month quantitative review of 189 patients, with 108 being in the intervention group, EMRs were analyzed for occurrence of infection. A 2-tailed T-test, assuming unequal variances, showed a mean rate of 0.11 infections per patient in the pre group and 0.03 in the post group (*p*=.065, 95% CI -0.007 to 0.0811). No adverse reactions were observed.

**Conclusions:**

PHBC was found to be non-inferior to CHG and is safe to use over burns, grafts, and intact skin. PHBC was not applied to skin substitutes in this study. Limitations of this pilot study are that only application of PHBC was considered. Other factors that could impact infection occurrence were not considered.

**Applicability of Research to Practice:**

PHBC is a safe alternative to CHG for skin and wound decolonization in patients with burn injuries. PHBC should be considered when selecting a skin antisepsis regimen in burn populations.

**Funding for the study:**

The research site was provided 3 months of free product by Avadim Health, Inc.

